# Seasonal Succession Leads to Habitat-Dependent Differentiation in Ribosomal RNA:DNA Ratios among Freshwater Lake Bacteria

**DOI:** 10.3389/fmicb.2016.00606

**Published:** 2016-04-29

**Authors:** Vincent J. Denef, Masanori Fujimoto, Michelle A. Berry, Marian L. Schmidt

**Affiliations:** Department of Ecology and Evolutionary Biology, University of MichiganAnn Arbor, MI, USA

**Keywords:** estuary, great lakes, potential activity, protein synthesis potential, particle-associated, free-living, 16S rRNA

## Abstract

Relative abundance profiles of bacterial populations measured by sequencing DNA or RNA of marker genes can widely differ. These differences, made apparent when calculating ribosomal RNA:DNA ratios, have been interpreted as variable activities of bacterial populations. However, inconsistent correlations between ribosomal RNA:DNA ratios and metabolic activity or growth rates have led to a more conservative interpretation of this metric as the cellular protein synthesis potential (PSP). Little is known, particularly in freshwater systems, about how PSP varies for specific taxa across temporal and spatial environmental gradients and how conserved PSP is across bacterial phylogeny. Here, we generated 16S rRNA gene sequencing data using simultaneously extracted DNA and RNA from fractionated (free-living and particulate) water samples taken seasonally along a eutrophic freshwater estuary to oligotrophic pelagic transect in Lake Michigan. In contrast to previous reports, we observed frequent clustering of DNA and RNA data from the same sample. Analysis of the overlap in taxa detected at the RNA and DNA level indicated that microbial dormancy may be more common in the estuary, the particulate fraction, and during the stratified period. Across spatiotemporal gradients, PSP was often conserved at the phylum and class levels. PSPs for specific taxa were more similar across habitats in spring than in summer and fall. This was most notable for PSPs of the same taxa when located in the free-living or particulate fractions, but also when contrasting surface to deep, and estuary to Lake Michigan communities. Our results show that community composition assessed by RNA and DNA measurements are more similar than previously assumed in freshwater systems. However, the similarity between RNA and DNA measurements and taxa-specific PSPs that drive community-level similarities are conditional on spatiotemporal factors.

## Introduction

High-throughput sequencing of conserved marker genes, particularly the small subunit ribosomal RNA gene, allows for rapid and affordable censuses of microbial life (Caporaso et al., 2012; Kozich et al., 2013). Increasingly, studies rely on marker gene assays that use RNA or both RNA and DNA (to calculate RNA:DNA ratios) as the input material based on the assumption that RNA or RNA:DNA ratio data better reflect active community members (e.g., Muttray and Mohn, 1999; Gentile et al., 2006; Jones and Lennon, 2010; Männistö et al., 2013; Zhang et al., 2014; Stibal et al., 2015). This is supported by better correspondence between key environmental drivers and RNA or RNA:DNA ratio marker gene data than DNA data (Hunt et al., 2013). RNA:DNA ratio data is particularly attractive as it normalizes RNA levels by a population's abundance. However, correlations between ribosomal RNA:DNA ratios and activity or growth rates are not always consistent (Blazewicz et al., 2013). For example, universal interpretations of growth rates or metabolic activities are confounded by the fact that such correlations may be different for organisms with different ecological strategies, such as oligotrophs and copiotrophs (Lankiewicz et al., 2015). Therefore, we have to be cautious how to interpret these data. A recent critical perspective has suggested to interpret the ribosomal RNA:DNA ratio of a bacterial population as the potential activity or even more conservatively, the protein synthesis potential (PSP) of a population (Blazewicz et al., 2013).

Depending on the environment, similarity between RNA and DNA based measurements of community composition can vary widely. In soils, community composition assessed using ribosomal RNA and DNA data tend to be highly divergent, which has been interpreted as a reflection of high levels of dormancy (Lennon and Jones, 2011; Baldrian et al., 2012; Barnard et al., 2013). In estuarine, coastal, and pelagic marine systems, community composition data from ribosomal RNA- and DNA-based surveys correspond more strongly than in soils, though for some populations these measurements are still significantly different (Lami et al., 2009; Campbell and Kirchman, 2013; Franklin et al., 2013; Hunt et al., 2013). Such differences in the relative abundance at the RNA and DNA level have been observed for both rare and abundant members of the community (Campbell et al., 2011; Hunt et al., 2013). For freshwater systems, weak correlation between abundance-weighted bacterial community composition assessed at the ribosomal RNA and DNA level was observed in the surface water of a set of north-temperate stratified lakes (Jones and Lennon, 2010). Thus, bacterial communities were more similar based on the surveyed nucleic acid type than based on the lake of origin. In addition, Jones and Lennon (2010) found that dormancy was more widespread among the bacterial community than the micro-eukaryotic community from the same samples in these systems. In contrast to some findings in soil, but similar to findings in marine systems, abundant freshwater taxa are generally detected and abundant in both RNA and DNA assays, whereas rare freshwater community members make up the majority of taxa unique to either assay (İnceoğlu et al., 2015).

A threshold of one for the ribosomal RNA:DNA relative abundance ratio has been used to differentiate “active” and “dormant” populations (Jones and Lennon, 2010). Limited data are available to validate this threshold, though one study reported that this method has led to two- to three-fold higher estimates for the fraction of active cells in marine estuarine environments compared to fluorescent staining techniques (Franklin et al., 2013). Much remains to be known about the factors that impact PSP (as inferred from ribosomal RNA:DNA ratios), including variation within and across phylogenetic groups, and across temporal and spatial gradients. Studies that have investigated this question along marine estuary to pelagic gradients have documented large changes in PSP for dominant populations in function of the salinity gradient (Campbell and Kirchman, 2013; Franklin et al., 2013). In addition, as the relative abundance of ribosomal RNA and DNA correlated more strongly in free-living communities relative to the entire community, PSPs of bacteria associated with particulate matter appear to be more variable across the spatial gradient than for free-living bacteria (Campbell and Kirchman, 2013).

In this study, we assessed the extent to which correlation between DNA and RNA measurements of bacterial community composition were affected by season, water column depth, and position along a freshwater estuarine to pelagic gradient in Lake Michigan, MI, USA. In addition, we identified taxa that drove community-level variation in PSP, measured by the 16S ribosomal RNA:DNA ratio, across space and time. Finally, we evaluated whether PSP is a phylogenetically conserved trait. While few data are available to allow for the extrapolation from PSP to ecosystem process contributions, the patterns we uncovered provide an important stepping-stone toward resolving the links between bacterial community composition, metabolic activity, and bacterially mediated ecosystem processes.

## Materials and methods

### Study site and sample collection

**Figure 1:**
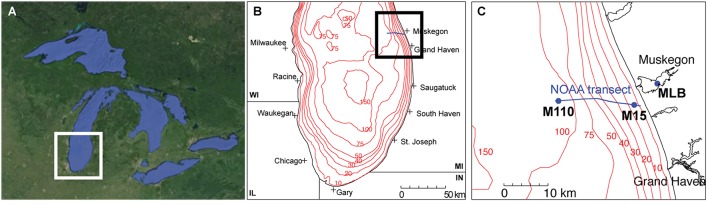
**Study sites. (A)** Regional map of the great Lakes (©Google Maps), **(B)** the southern basin of Lake Michigan, and **(C)** the locations of the stations along the Muskegon NOAA long-term research transect [blue line in **(B,C)**] at which samples were collected (MLB, Muskegon Lake buoy site; M15, Lake Michigan 15 m depth station; M110, Lake Michigan 110 m depth station).

We collected all water samples aboard the R/V Laurentian during the NOAA Great Lakes Environmental Research Laboratory spatial surveys on Lake Michigan near Muskegon, MI, USA in April, July, and September 2013 (Figure 1; Table 1). Three stations were included: (a) the Muskegon Lake monitoring buoy (www.gvsu.edu/wri/buoy/), a eutrophic drowned river mouth freshwater estuary (Steinman et al., 2008) (MLB; 43° 14′ 17″ N, 86° 16′ 49″ W), (b) the 15 m water column depth near-shore station on oligotrophic Lake Michigan (M15; 43° 11′ 17″ N, 86° 20′ 38″ W) and the 110 m water column depth off-shore station on oligotrophic Lake Michigan (M110; 43° 11′ 59″ N, 86° 34′ 11″ W) (Vanderploeg et al., 2010; Denef et al., 2016). Water was collected at the surface (5 m below surface in Lake Michigan, 1–2 m below in Muskegon Lake), and deep (5 m above lake floor in Lake Michigan, 2 m above in Muskegon Lake) with the exception of spring MLB, where we only collected one sample at 5 m depth (labeled surface), and summer M110, where we also sampled at the chlorophyll maximum (36 m below surface). The surface was sampled during the day and night, except for MLB, where we only sampled once. This resulted in 24 total sampling events (Table 1). For each sampling event we collected water using a 30 L Niskin bottle, which was pre-filtered through 210 and 20 μm nitex mesh and collected into 10 L carboys. All sampling gear except the 30L niskin bottle were bleach-cleaned and rinsed with MilliQ water until no odor remained before each sampling cruise, and rinsed twice with sample water right before sampling. We divided pre-filtered water in two fractions by sequentially filtering onto 3.0 μm isopore polycarbonate filters [particulate (PA) fraction] and 0.22 μm [free-living (FL) fraction] polyethersulfone filter membranes (142 mm, Millipore) using a Masterflex I/P peristaltic pump (Cole Parmer) between settings 11 and 13. Size fractionation doubled our sample total to 48. We limited filtering to 10 min and submerged folded filters in RNAlater (Ambion) within 20 min of sampling. Filtered water volumes are reported in Table 1. Filters were stored at −20°C on board and transported on dry ice to a −80°C freezer within 2 days of sampling.

**Table 1:** **Sample events**.

**Sampling event**	**Successful sequencing libraries**	**Collection date**	**Collection time**	**Station**	**Sampling depth (m)**	**Water volume (L)**
Sp13.M15.SN	FL-D, FL-R, PA-D (1), PA-R	Apr 24, 2013	1:10 a.m.	M15	5	4.0
Sp13.M15.SD	FL-D (1), FL-R, PA-D, PA-R	Apr 23, 2013	10:40 a.m.	M15	5	7.5
Sp13.M15.DD	FL-D (1), FL-R, PA-D, PA-R	Apr 23, 2013	1:30 p.m.	M15	10	9.5
Sp13.M110.SN	FL-D, FL-R, PA-D (1), PA-R	Apr 23, 2013	10:00 p.m.	M110	5	7.5
Sp13.M110.SD	FL-D, FL-R, PA-D, PA-R	Apr 23, 2013	5:45 p.m.	M110	5	8.5
Sp13.M110.DD	FL-D (1), FL-R, PA-D (1), PA-R	Apr 23, 2013	6:36 p.m.	M110	108	9.0
Sp13.MLB.SN	FL-D, FL-R, PA-D (1), PA-R	Apr 24, 2013	2:09 a.m.	MLB	5	2.8
Su13.M15.SN	FL-D, FL-R, PA-D, PA-R	July 15, 2013	9:25 p.m.	M15	5	10.0
Su13.M15.SD	FL-D, FL-R, PA-D, PA-R	July 16, 2013	2:35 p.m.	M15	5	10.0
Su13.M15.DN	FL-D, FL-R, PA-D, PA-R	July 15, 2013	10:00 p.m.	M15	15	10.0
Su13.M110.SN	FL-D, FL-R, PA-D, PA-R	July 16, 2013	3:25 a.m.	M110	5	10.0
Su13.M110.SD	FL-D, FL-R, PA-D, PA-R	July 16, 2013	8:41 a.m.	M110	5	10.0
Su13.M110.DN	FL-D, FL-R, PA-R	July 16, 2013	4:03 a.m.	M110	110	10.0
Su13.M110.DCMD	FL-D, FL-R, PA-D, PA-R	July 16, 2013	9:20 a.m.	M110	36	10.0
Su13.MLB.SD	FL-D (1), FL-R (1), PA-D, PA-R	July 15, 2013	6:15 p.m.	MLB	1	10.0
Su13.MLB.DD	FL-D (1), FL-R (1), PA-D, PA-R	July 15, 2013	6:55 p.m.	MLB	8	8.5
Fa13.M15.SN	FL-D, FL-R, PA-D, PA-R	Sep 23, 2013	9:18 p.m.	M15	5	11.0
Fa13.M15.SD	FL-D, FL-R, PA-D, PA-R	Sep 24, 2013	1:30 p.m.	M15	5	9.0
Fa13.M15.DN	FL-D, FL-R, PA-D, PA-R	Sep 23, 2013	10:00 p.m.	M15	15	10.0
Fa13.M110.SN	FL-D, FL-R, PA-D, PA-R	Sep 24, 2013	3:04 a.m.	M110	5	10.0
Fa13.M110.SD	FL-D, FL-R, PA-D, PA-R	Sep 24, 2013	9:28 a.m.	M110	5	10.5
Fa13.M110.DN	FL-D, FL-R, PA-D, PA-R	Sep 24, 2013	4:02 a.m.	M110	108	10.5
Fa13.MLB.SN	FL-D (1), FL-R (1), PA-D, PA-R (1)	Sep 23, 2013	7:45 p.m.	MLB	2	10.0
Fa13.MLB.DN	FL-D (1), FL-R (1), PA-D, PA-R	Sep 23, 2013	8:10 p.m.	MLB	8	10.0

For each sampling event, free-living (FL; 0.22–3 μm) and particulate (PA: 3–20 μm) fractions were collected, from which DNA (D) and RNA (R) was simultaneously extracted and submitted for 16S rRNA gene V4 region sequencing. Samples for which only one replicate dataset was available are marked with (1).

### DNA/RNA extraction and sequencing

We performed duplicate nucleic acid extractions from the same 142 mm filter membrane for each of the field samples, which resulted in 96 samples total. We simultaneously extracted RNA-free DNA and DNA-free RNA using a modified AllPrep DNA/RNA/miRNA Universal kit protocol (Qiagen) (McCarthy et al., 2015). We converted 2 μl of each RNA fraction (out of ~30 μl total) to cDNA using the ProtoScript II First Strand cDNA Synthesis Kit (New England BioLabs). The DOE Joint Genome Institute carried out amplicon sequencing targeting the V4 region of the 16S rRNA gene (515F/806R universal primers) (Caporaso et al., 2012). After omission of one replicate for the summer and fall MLB samples to fit the assay number limitation, 92 RNA (cDNA) libraries (run 1; 91 successful, Table 1), and 92 DNA libraries (run 2; 83 successful, Table 1) were sequenced on an Illumina MiSeq sequencer, using v2 chemistry 2 × 250 (500 cycles) paired-end reads. RTA v1.17.28 and MCS v2.2.0 software were used to generate data. To reduce computational time, we processed a random subset of 40,000 quality-filtered read pair contigs (quality filtering performed by JGI's iTagger pipeline). We used mothur v.1.34.3 to generate the operational taxonomic unit (OTU, 97% sequence similarity) table. We used the MiSeq standard operating protocol accessed on Dec 17, 2014 using SILVA release 119 for alignment and OTU generation (Schloss et al., 2011; Quast et al., 2013). Classification was based on a hybrid protocol using a freshwater-specific taxonomy (https://github.com/mcmahon-uw/FWMFG) and the SILVA release 119 taxonomy as previously described (Schmidt et al., 2016). We removed all non-bacterial and chloroplast sequences from the analysis. All data is available on the Joint Genome Institute's genome data portal (http://genome.jgi.doe.gov/; Project IDs 1041195 and 1041198). Mothur output files, classification files and procedure are available at https://github.com/DenefLab/Frontiers2016Denef and the procedure is also included as an R markdown document (Supplemental File 1).

### Statistical analyses

All subsequent analyses were performed in R version 3.2.1 using the phyloseq (McMurdie and Holmes, 2013), vegan (Oksanen et al., 2013), and ggplot2 R packages (Wickham, 2009). Full code and input files are available at https://github.com/DenefLab/Frontiers2016Denef and as an R markdown document (Supplemental File 1).

To determine differences in bacterial community composition, we merged replicate samples by averaging, rounding down, and scaling each OTU read count to the smallest merged library size (4609 sequences, as some samples resulted in >80% chloroplast sequences) (McMurdie and Holmes, 2014). We calculated the (abundance-weighted) Bray-Curtis dissimilarity between samples, and generated a dendrogram representing sample similarity by UPGMA hierarchical clustering similar to (Figure 1 in Jones and Lennon, 2010). Symbols and line thickness were adjusted in Illustrator (Adobe, Inc.). We performed a nested permutational multivariate analysis of variance (PERMANOVA; Anderson, 2005) using the *adonis* function (vegan) to test if season, filter fraction, lake, station, depth, and day/night could significantly explain variation in the bacterial community composition. We also performed a partial Mantel test (*mantel.partial* function in vegan) to determine whether environmental factors explained additional variation in RNA level Bray-Curtis dissimilarities after DNA-level variation had been taken into account.

The number of OTUs that were shared between matching DNA-RNA samples and between technical replicates was calculated using the *intersect* function in base R. This analysis was performed for the OTUs retained after sample merging (which resulted in retaining of 2211 OTUs) as well as for the top 200 most abundant OTUs. To test for significant differences in OTU overlap based on season, sampling location, filter fraction, or depth, we performed a Kruskall-Wallis test (*kruskal.test*; R Core Team., 2015) along with *post-hoc* tests to identify significant pairwise differences (*kruskalmc* in pgirmess R-package; Giraudoux, 2012), which were visualized using the *multcompLetters* function (multcompView R-package; Graves et al., 2012).

To identify significant differences in the contribution to the RNA and DNA pool by specific taxa, we used the negative binomial generalized linear model framework of the *DESeq* function in the DESeq2 R-package (Love et al., 2014; McMurdie and Holmes, 2014). *P*-values were adjusted for multiple testing through the Benjamini-Hochberg false discovery rate correction (Love et al., 2014).

We calculated the Euclidean distance between samples based on the log_2_ ribosomal RNA:DNA ratios and visualized these distances in Principal Coordinates Analysis (PCoA) ordination using the *pcoa* function (vegan). Similarly, we calculated the Bray-Curtis dissimilarity among RNA samples and among DNA samples and visualized these in a PCoA ordination. A Kruskall-Wallis test was performed as described above to compare whether sample dissimilarity in ribosomal RNA:DNA ratios between PA and FL, Muskegon Lake and Lake Michigan, and surface and deep water was a function of season. We performed a PERMANOVA analysis as described above to determine if season, filter fraction, lake, station, and day/night could significantly explain variation in log_2_ ribosomal RNA:DNA ratios between samples. Finally, we performed a Procrustes analysis (*procrustes* and *protest* functions in vegan) to determine whether the ordination patterns were significantly different between DNA, RNA, and RNA:DNA ratio data.

To identify the taxa that were drivers of community-level similarities in ribosomal RNA:DNA ratios between FL and PA fraction in spring, and differences in summer and fall, we identified OTUs that did not have differences in ribosomal RNA:DNA ratio between spring Lake Michigan PA and FL fractions (Wilcoxon test, *P* > 0.10), but did have significant differences in ribosomal RNA:DNA ratio between summer and fall Lake Michigan PA and FL fractions (Wilcoxon test, Benjamani Hochberg false discovery rate adjusted *P* < 0.01).

## Results

**Figure 2:**
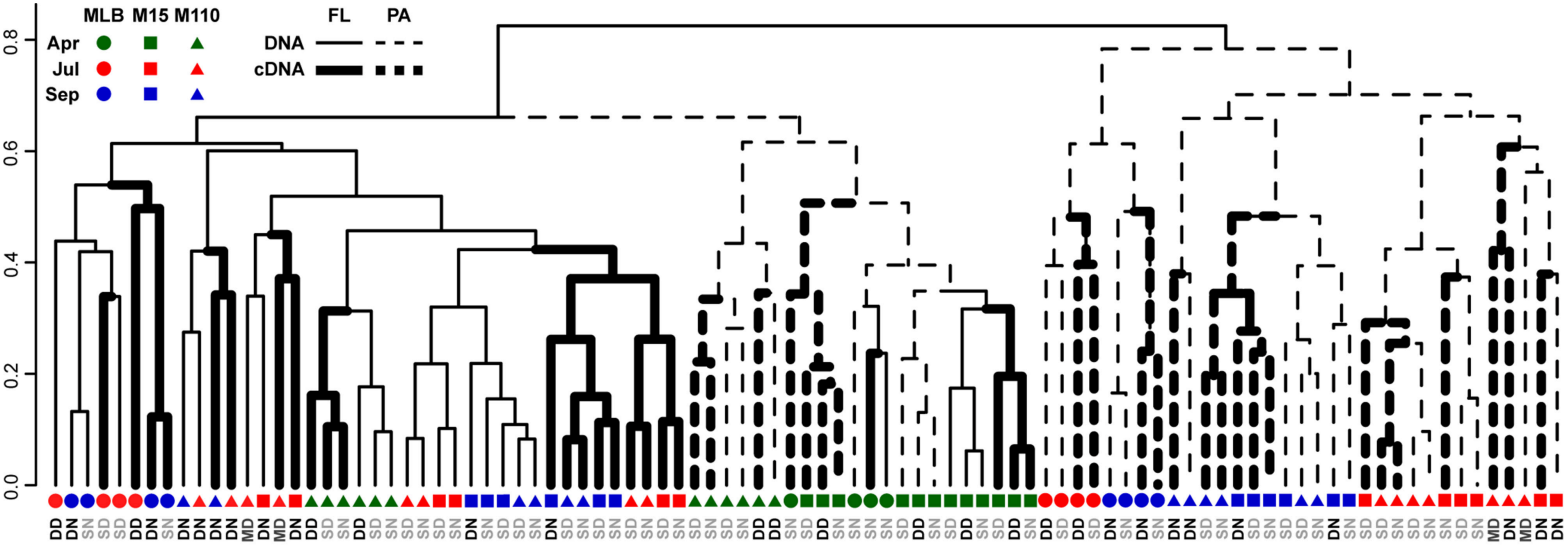
**Dendrogram reflecting similarity between DNA and RNA level bacterial community composition**. UPGMA hierarchical clustering based on abundance-weighted bacterial community dissimilarity (Bray-Curtis). Community composition data included the 2211 OTUs retained after replicate sample merging (averaging with rounding down) and scaling of the data to the smallest dataset size. Letter colors discriminate surface (gray) from deep and chlorophyll maximum (black) water samples: DN, Deep, nighttime; DD, Deep, daytime; SN, Surface, nighttime; SD, Surface, daytime; MD, Chlorophyll maximum, daytime.

Groups of samples clustered primarily by filter fraction [free-living (FL) vs. particulate (PA)], time, and station [particularly Lake Michigan (M15 and M110) vs. Muskegon Lake estuary (MLB) samples; Figure 2]. The effect of nucleic acid type (16S rRNA gene sequencing of DNA or RNA samples) typically affected community composition less than these other factors. In other words, community composition in DNA and RNA samples taken at the same time, location, and fraction were often the most similar (Figure 2). However, there were exceptions to this general trend. Most prominently, all surface FL RNA samples from nearshore and offshore Lake Michigan communities during the stratified period (summer and fall) clustered with each other, while DNA samples from the same locations and time formed a separate cluster. This pattern was less pronounced for the corresponding PA samples, as samples from the same season and sampling site (in summer) clustered more closely together independent of nucleic acid type. The clustering patterns were supported by PERMANOVA analysis that indicated variation among abundance-weighted community composition could be explained primarily by filter fraction, season, and lake/station (Table 2, “RNA, DNA”). Nucleic acid type was a significant factor affecting community composition, but explained only a small proportion of the variation (a similar amount as sampling depth). When taking into account between-sample dissimilarities observed at the DNA level, additional variation at the RNA level still significantly correlated to environmental factors (fraction, season, lake, station, sampling depth, and day vs. night; Mantel statistic *r* = 0.26, *p* = 0.001). However, the correlation was weaker relative to the correlation between the RNA level Bray-Curtis dissimilarity and the same environmental factors without accounting for the patterns already observed at the DNA level (*r* = 0.51, *p* = 0.001). When repeating the latter analysis one environmental factor at a time, only “Season” and “Lake” resulted in significant partial Mantel test statistics (*p* < 0.05).

**Table 2:** ***R*^2^ values and *p*-values for nested PERMANOVA analysis of relative abundance (RNA, DNA) and ratio data (RNA:DNA)**.

**Factor**	**Fraction**	Season^*^	Lake^**^	**Station**	Depth^***^	**Nucleic acid**	**Diel**	**Residuals**
Values	*PA, FL*	*Sp, Su, Fa*	*ML, LM*	*MLB, M15, M110*	*S, M, D*	*DNA, RNA*	*Day, night*	–
RNA (*n* = 48), DNA (*n* = 47)	0.22 (0.001)	0.18 (0.001)	0.07 (0.001)	0.03 (0.001)	0.05 (0.001)	0.05 (0.001)	0.01 (0.06)	0.41
RNA:DNA (*n* = 47)	0.17 (0.001)	0.15 (0.001)	0.09 (0.001)	0.04 (0.008)	0.05 (0.02)	– (–)	0.01 (0.59)	0.58

* Sp, spring; Su, summer; Fa, fall;

** ML, Muskegon Lake; LM, Lake Michigan;

*** *S, surface; M, chlorophyll maximum; D, deep*.

**Figure 3:**
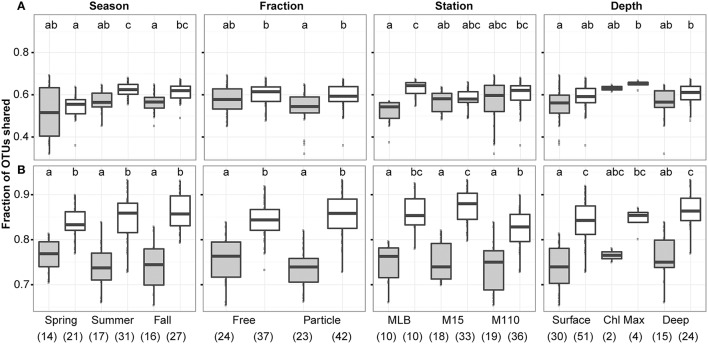
**Overlap of observed OTUs between samples in function of season, filter fraction, station, and depth**. Boxplots show overlap between RNA and DNA level data (gray) in contrast to the overlap between replicate samples (both DNA and RNA data; white). Analysis was performed using **(A)** 2211 OTUs retained after replicate sample merging and scaling to the smallest dataset size and **(B)** the top 200 most abundant OTUs across. In each panel, letter(s) above boxplots differentiate sample groups that are significantly different in their degree of OTU overlap (i.e., categories with significantly different OTU overlap do not share any letters), as determined by pair-wise *post-hoc* testing of the Kruskall-Wallis non-parametric one-way ANOVA test results. Numbers in parentheses below the x-axis labels represent the number of comparisons included for each sample grouping. Comparisons always excluded self-comparisons.

We did not observe a difference in the proportion of OTUs that were identified by both DNA and RNA assays between seasons, filter fractions, stations, or depths (Figure 3). However, we did observe some differences in the proportion of OTUs identified between replicate samples and paired RNA and DNA samples. When including all 2211 OTUs identified after averaging replicates and scaling all averaged data sets to the smallest merged library size (4609 sequences), overlap between OTUs identified in the RNA and DNA samples were significantly lower than between replicate sample overlap in summer and fall, for PA communities, and at the Muskegon Lake station. When using only the top 200 most abundant OTUs, all sample groups displayed a lower proportion of shared OTUs between RNA and DNA samples as compared to replicate samples (Figure 3). The top 200 OTUs corresponded to the OTUs for which on average at least two sequences per sample were present after averaging and scaling. We focused the remainder of our analyses on this reduced dataset of the 200 most abundant OTUs. These 200 OTUs contribute on average to 91 ± 7% of sequences per sample when including all 2211 OTUs.

**Figure 4:**
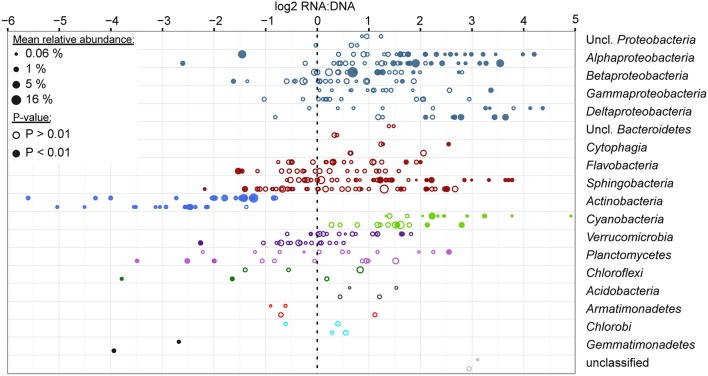
**Ratio of relative abundance in RNA and DNA level analyses for the top 200 most abundant OTUs**. Analyses were performed using DEseq2 for FL and PA datasets separately (top and bottom data rows for each phylum/class, respectively), combining data across all seasons, stations, depths and time of sampling for each OTU. Each circle represents an OTU and is scaled based on the average relative abundance of the OTU in the respective filter fraction. Filled circles represent OTUs that were significantly differentially represented between RNA and DNA (*P*-value adjusted for multiple testing < 0.01). Data are sorted by average relative abundance of each phylum across all samples and are colored by phylum. *Proteobacteria* and *Bacteroidetes* were split up into classes due to the high number of OTUs in these groups.

Among abundantly represented phyla, *Actinobacteria* and to a lesser extent particulate fraction *Planctomycetes* contributed significantly less to the community RNA pool than to the DNA pool (Figure 4). *Alphaproteobacteria* (except for tribe LD12), free-living *Betaproteobacteria*, and most *Cyanobacteria* tended to contribute more to the community RNA pool than to the DNA pool. Particulate fraction *Betaproteobacteria*, and several *Bacteroidetes* classes showed either limited differential representation between observed RNA and DNA community composition, or displayed a mixed signal (Figure 4). As *Actinobacteria* contribute to a larger fraction of FL than PA communities, their low ribosomal RNA:DNA ratios in both fractions led to most other groups displaying higher average FL than PA ribosomal RNA:DNA ratios. When removing *Actinobacteria* from the analysis, this shift indeed disappears, though similar groups remain over- and underrepresented in the RNA and DNA pool (data not shown). The most over- and/or underrepresented OTUs in both fractions were listed in Table 3.

**Table 3:** **OTUs with largest and smallest log_2_ fold RNA:DNA ratios (Δ) in FL and PA fractions**.

**Phylum/Class (OTU)**	**Order**	**Family**	**Genus (Tribe)**	**FL**		**PA**	
				**%**	**Δ (P)**	**%**	**Δ (P)**
**HIGH RIBOSOMAL RNA:DNA RATIO**							
*Alphaproteobacteria* (133)	*Rhodospirillales*	*Acetobacteraceae*	–	0.191	4.2 (^***^)	0.046	2.87 (^*^)
*Alphaproteobacteria* (30)	*Caulobacterales*	*Hyphomonadaceae*	*Hirschia*	0.148	3.21 (^***^)	3.877	3.54 (^***^)
*Betaproteobacteria* (139)	*Burkholderiales*	*Oxalobacteraceae*	–	0.024	2.74 (^*^)	0.167	0.96 ()
*Betaproteobacteria* (78)	*Burkholderiales*	*Burkholderiaceae*	–	0.341	1.63 ()	0.166	1.65 (^*^)
*Gammaproteobacteria* (55)	*Methylococcales*	*Methylococcaceae*	*Methylobacter*	0.408	3.36 (^*^)	0.360	1.92 ()
*Deltaproteobacteria* (41)	*Bdellovibrionales*	*Bdellovibronaceae*	OM27	0.066	4.36 (^***^)	1.494	3.65 (^***^)
*Sphingobacteria* (65)	*Sphingobacteriales*	–	–	0.077	3.71 (^***^)	0.889	2.51 (^***^)
*Cytophagia* (90)	*Cytophagales*	–	–	0.112	2.54 (^***^)	1.453	2.06 ()
*Flavobacteria* (235)	*Flavobacteriales*	bacV	–	0.078	2 (^***^)	0.162	0.92 ()
*Sphingobacteria* (25)	*Sphingobacteriales*	bacVI	–	0.085	3.77 (^***^)	1.544	2.67 ()
*Sphingobacteria* (72)	*Sphingobacteriales*	bacVI	–	0.071	3.65 (^***^)	0.502	2.47 (^*^)
*Cyanobacteria* (39)	Subsection I	Family I	*Microcystis*	0.003	4.91 (^***^)	0.821	2.8 (^*^)
*Verrucomicrobia* (49)	*Opitutales*	*Opitutaceae*	–	0.319	1.63 (^*^)	0.160	−0.11 ()
*Planctomycetes* (34)	*Phycisphaerales*	*Phycisphaeraceae*	CL500-3	0.567	2.55 (^***^)	1.923	1.52 ()
**LOW RIBOSOMAL RNA:DNA Ratio**							
*Alphaproteobacteria* (9)	*Rickettsiales*	alfV	alfV-A (LD12)	5.176	−1.45 (^**^)	0.283	−2.6 (^***^)
*Betaproteobacteria* (33)	*Methylophilales*	betIV	betIV-A (LD28)	2.512	−0.05 ()	0.274	−1.62 (^*^)
*Flavobacteria* (16)	*Flavobacteriales*	bacV	–	0.616	−0.49 ()	1.599	−1.53 (^*^)
*Sphingobacteria* (291)	*Sphingobacteriales*	bacVI	–	0.100	−0.25 ()	0.024	−2.18 (^*^)
*Actinobacteria* (318)	*Acidimicrobiales*	acIV	acIV-A (Iluma-A2)	0.173	−5.6 (^***^)	0.045	−5.03 (^***^)
*Planctomycetes* (359)	*Planctomycetales*	*Planctomycetaceae*	–	0.038	−2.22 ()	0.123	−3.49 (^***^)
*Chloroflexi* (765)	SL56 marine group	–	–	0.248	−1.39 ()	0.046	−3.78 (^***^)
*Verrucomicrobia* (26)	*Opitutales*	*Opitutaceae*	–	1.025	0.04 ()	0.357	−2.26 (^*^)
*Gemmatimonadetes* (145)	*Gemmatimonadales*	*Gemmatimonadaceae*	*Gemmatimonas*	0.063	−2.68 (^***^)	0.141	−3.94 (^***^)

Top OTUs were included for each phylum and Proteobacteria and Bacteroidetes class that contained OTUs among the top 200 most abundant taxa that were differentially represented in RNA and DNA data. We also reported the overall relative abundance of the OTUs across all FL or PA datasets (%) and the adjusted P-values reported by DEseq2 [ < 0.01 (^*^), < 0.001 (^**^), < 0.0001 (^***^)].

**Figure 5:**
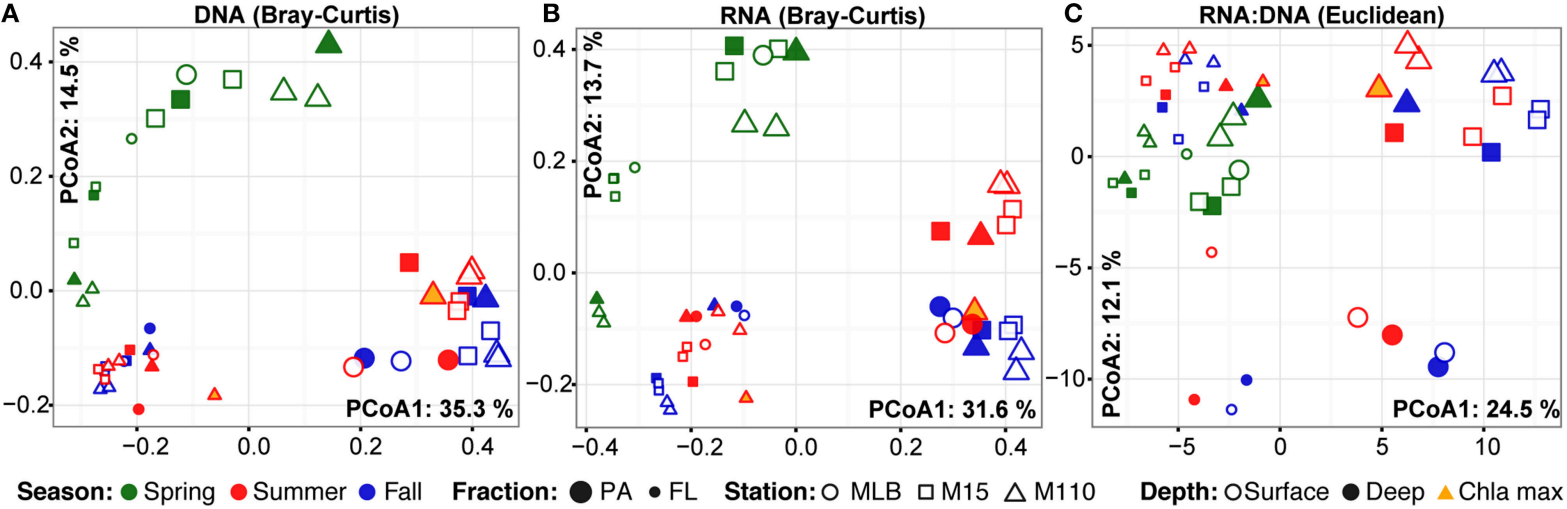
**PCoA ordination of RNA and DNA level community composition and ribosomal RNA:DNA ratios**. First two axes of the principal coordinates analysis representing the Bray-Curtis dissimilarity between **(A)** DNA level community composition and **(B)** RNA level community composition. In addition, **(C)** represents the PCoA based on the Euclidean distance between samples based on log_2_(RNA:DNA) data. All ordinations are based on analyses including the top 200 most abundant OTUs. Axes labels include the % variation captured by this dimension of the ordination. PA (large) and FL fractions (small) were differentiated by glyph size.

**Figure 6:**
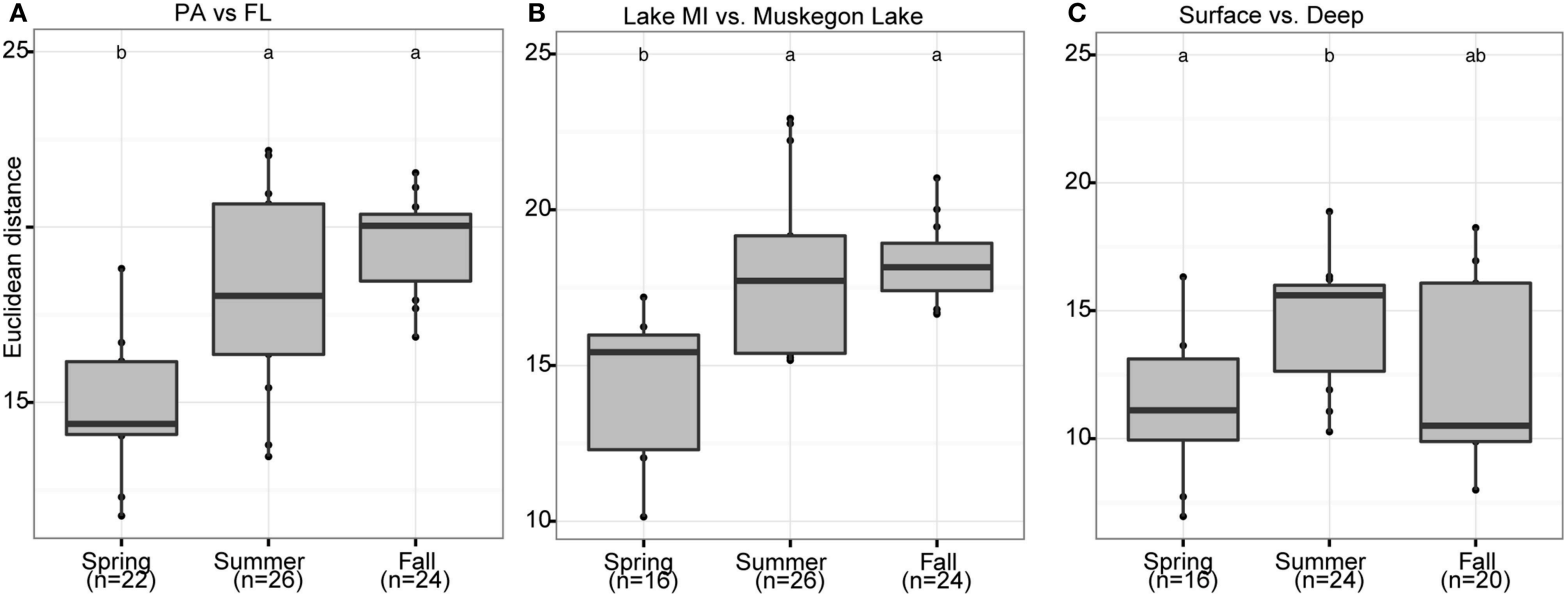
**Seasonal shifts in habitat-dependent RNA:DNA ratios**. Boxplots representing the Euclidean distance (Y-axis) between samples based on log_2_(RNA:DNA) data when considering the 200 most abundant OTUs. The boxplots display seasonal differences between RNA:DNA ratios of the community present in different habitats: **(A)** PA and FL fractions, **(B)** Lake Michigan relative to Muskegon Lake, and **(C)** surface compared to bottom waters. In each panel, letter(s) above boxplots differentiate sample groups that have significantly different Euclidean distances between habitats across seasons, as determined by pair-wise *post-hoc* testing of the Kruskall-Wallis non-parametric ANOVA test results. Numbers in parentheses below the x-axis labels represent the number of pair-wise habitat comparisons for each season.

**Figure 7:**
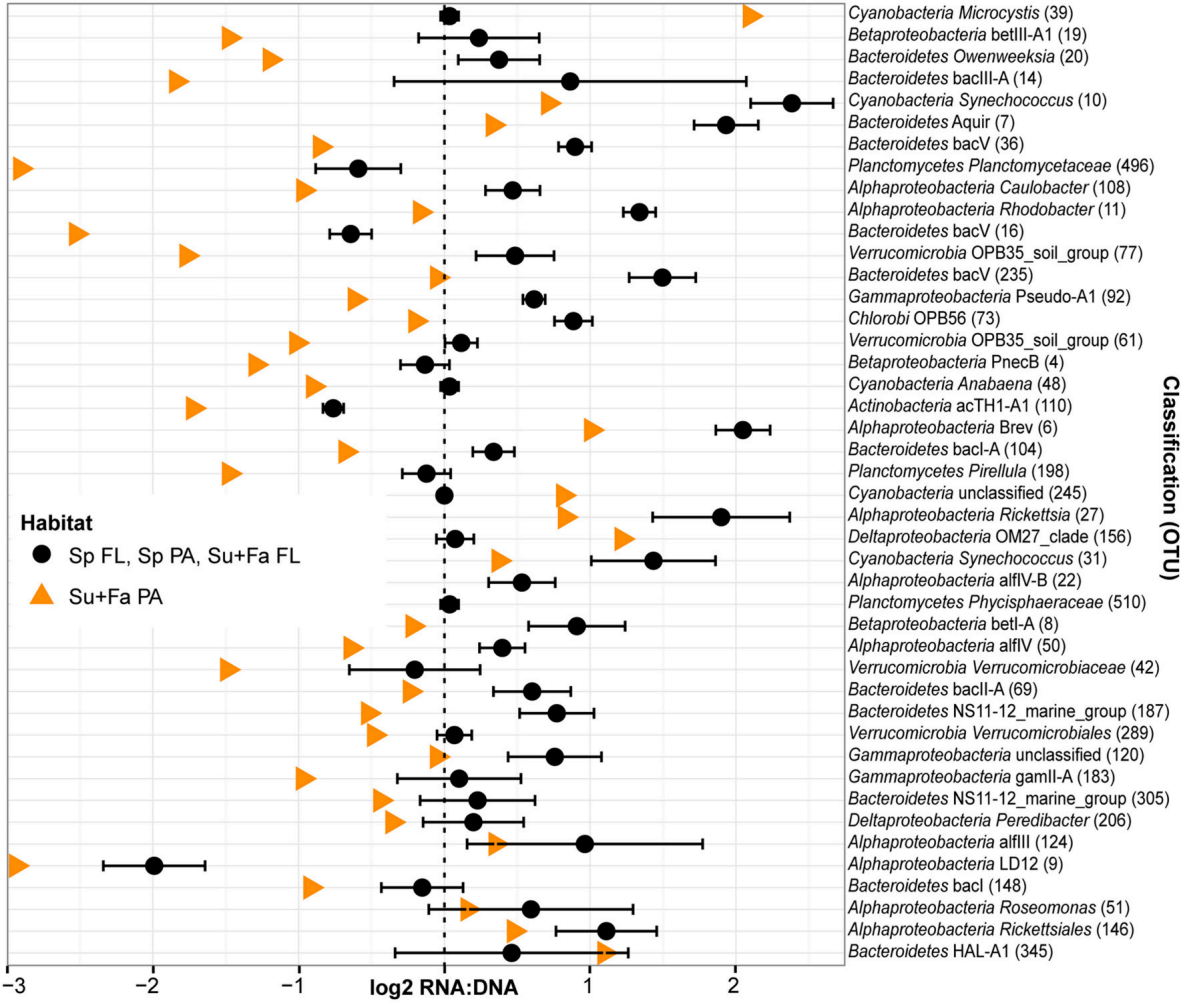
**Taxa driving the similarity between spring PA and FL ribsosomal RNA:DNA ratios relative to summer and fall**. OTUs that did not have significantly different mean RNA:DNA ratios between PA and FL communities in spring (Wilcoxon test, *P* > 0.10) but did have significantly different mean ratios in summer and fall (Wilcoxon test, false discovery rate adjusted *P* < 0.01). Cutoff stringency was set at different levels as the number of replicates was twice as high for the combined summer and fall analysis relative to the spring analysis. As community wide ribosomal RNA:DNA ratios for FL and PA fractions in spring and FL fraction in summer and fall were similar to each other, we averaged these three values (black circles; error bars indicate standard deviation between Sp FL, Sp PA, and Su+Fa FL). Data for Sp FL, Sp PA, Su+Fa PA, and Su+Fa FL was obtained by averaging the log_2_ RNA:DNA ratio data across sampling sites. Analysis included only the top 200 most abundant OTUs and excluded data from Muskegon Lake. Taxa are sorted based on the difference in ribosomal RNA:DNA ratio between spring and summer/fall PA fraction relative to the difference between ribosomal RNA:DNA ratio between spring PA and FL fractions. In addition to the OTU taxonomy, the OTU number was added between parentheses.

Season, fraction, and lake were correlated most strongly with variation in community wide OTU-level RNA:DNA ratios across space and time (Table 2, “RNA:DNA” row). DNA and RNA sample community composition ordination patterns were correlated more strongly along the first two dimensions of the PCoA than compared to the RNA:DNA ratio ordination (Figures 5A–C; Procrustes correlations DNA-RNA = 0.96, DNA-RNA:DNA = 0.67, RNA-RNA:DNA = 0.68, all correlations were significant at *p* = 0.001). Most noticeably, while the RNA and DNA spring samples clustered separately from the summer and fall samples and PA and FL communities formed separate clusters, both PA and FL spring RNA:DNA ratio data clustered together with FL Lake Michigan summer and fall communities. In an alternate approach to analyze these patterns, we observed that the distance between community-level RNA:DNA ratios in PA relative to FL fractions, in Lake Michigan relative to Muskegon Lake, and in surface relative to deep water was generally lower in spring relative to summer and fall (Figure 6). In line with the clustering of spring PA and FL samples across seasons, we identified a series of OTUs that display similar ribosomal RNA:DNA ratios in spring PA and FL and summer and fall FL fractions, but significantly different ratios in PA summer and fall communities (Figure 7). Mostly, these included taxa with higher RNA:DNA ratios in spring PA and all FL fractions relative to summer/fall PA fractions, except for a *Microcystis* OTU that was much more abundant and had a higher RNA:DNA ratio in summer and fall. As Muskegon Lake samples showed distinct RNA:DNA ratio patterns from Lake Michigan samples (Figure 5), we excluded these samples for the analysis in Figure 7.

## Discussion

This study builds on previous findings in aquatic and terrestrial systems regarding discrepancies in bacterial community composition observed when using matching environmental DNA and RNA extracts. Similar to previous studies, we observed differences between DNA and RNA measurements of community composition with respect to OTU identity and relative abundance. Yet, we showed higher correspondence between RNA and DNA measurements of community composition relative to changes across spatiotemporal gradients. This result, which contrasts previous work in freshwater systems, can most likely be explained by the inclusion of larger environmental gradients compared to previous work. Our study also revealed seasonal patterns in the similarity of ribosomal RNA:DNA ratios across habitats (defined by filter fraction, water column depth, and the location on the estuary to pelagic gradient). In spring, we observed higher between-habitat similarities in RNA:DNA ratios than in summer and fall. Finally, we contribute to the expanding work on analyzing the phylogenetic conservation of traits by revealing how PSP, as measured by ribosomal RNA:DNA ratios, is conserved for some phyla, most likely as a function of cell size.

Our observation that sample community composition varies more across spatiotemporal gradients than between RNA and DNA measurements opposes previous analyses by Jones and Lennon (2010). They showed that bacterial communities in surface water samples collected in September 2008 from eight inland lakes of varying trophic states (oligotrophic to eutrophic; >0.22 micron fraction) formed two main clusters: one containing all RNA measurements and one containing all DNA measurements. In contrast, our analyses included more stark environmental gradients, capturing particularly large changes in community composition across seasons and size fractions that explained more of the community composition variation than nucleic acid type did. However, for samples most closely resembling those taken by Jones and Lennon (i.e., surface free-living communities sampled during the stratified period in July and September 2013), we did observe similar subclusters of DNA-only and RNA-only measurements in Lake Michigan.

In Muskegon Lake there was less overlap between OTUs observed in the RNA and DNA measurements than between replicate DNA or replicate RNA samples. This result may be explained by higher bacterial richness in urban estuaries due to urban runoff and influx of riverine and soil bacteria (Newton and McLellan, 2015). Additionally, Muskegon Lake has a relatively low residence time (~3 weeks; Steinman et al., 2008) compared to Lake Michigan (~60 years; Quinn, 1992). These two factors lead to a higher impact of mass effects relative to local species sorting in Muskegon Lake, and thus, maladapted bacterial cells that are no longer alive or active may be detected with DNA-based methods (Adams et al., 2014).

Using similar reasoning, we can explain differences in RNA and DNA measurements for PA fractions. Particles transport bacterial communities horizontally and vertically through aquatic systems (Fontanez et al., 2015). Therefore, members of PA communities are more likely to be maladapted to current conditions than free-living populations. This would lead to a larger number of dead or dormant cells in PA fractions, explaining the lower overlap between RNA and DNA measurements. In fact, lower correspondence between OTUs in matching DNA and RNA measurements from PA fractions relative to FL fractions has been documented in marine systems as well (Ghiglione et al., 2009). Overall, our observation of higher overlap between abundant OTUs in paired DNA and RNA samples supports previous work showing that most unique OTUs from DNA or RNA measurements of the same sample are rare (İnceoğlu et al., 2015).

We used two methodologies in this study that were important for eliminating biases that might confound differences in RNA and DNA measurements of community composition. First, we took replicate samples (separate parts of the same 142 mm filter, extracted and sequenced separately on the same sequencing run). This allowed us to evaluate the impact of stochastic effects associated with sampling and sequencing on OTU overlap. Subsequently, we contrasted DNA and RNA OTU overlap to replicate sample overlap. This allowed us to identify significant impacts of habitat on DNA and RNA sample OTU overlap that we could not have made without the availability of replicate measurements. We also reduced the impacts of stochasticity by averaging OTU counts from replicates and scaling the read depth to the smallest merged library size to remove the impact of many rare and spurious OTUs (McMurdie and Holmes, 2014). Zhou et al. (2011) have previously reported on the influence of these stochastic effects on OTU identification. They observed < 20% overlap in detected OTUs between repeated 16S rRNA gene sequencing from the same soil DNA extract (Zhou et al., 2011).

The second method we used to reduce confounding factors when comparing RNA and DNA measurements was the simultaneous extraction of RNA and DNA from one sample (Morgan et al., 2010; McCarthy et al., 2015). While RNA and DNA libraries were sequenced on a separate run, this tends to introduce much less variation than extraction differences (Schloss et al., 2011; McCarthy et al., 2015). The potential impact of using simultaneous vs. independent DNA and RNA extractions is exemplified by the RNA:DNA ratios we observed for *Verrucomicrobia*. A previous study using separate DNA and RNA extraction protocols on freshwater reservoir samples indicated that *Verrucomicrobia* are disproportionately inactive in freshwater systems relative to their abundance (Tsementzi et al., 2014), but our data does not support this claim. Other factors that may contribute to differences with the Tsementzi study (2014) are system-dependent factors and the use of metatranscriptomics data instead of rRNA gene data.

Among bacteria, phylum and class level conservation of traits generally appears to be limited (Martiny et al., 2013). Ribosomal RNA:DNA ratios are an indicator of the number of ribosomes carried per cell and can be interpreted conservatively as the PSP (Blazewicz et al., 2013). While we observed a range of ribosomal RNA:DNA ratios at the class and phylum level, tendencies of over- and underrepresentation in RNA relative to DNA measurements were consistent for several phyla or classes. In contrast, the wide spectrum of RNA:DNA ratios observed for *Bacteroidetes* is consistent with previous observations for other traits of this phylum, such as habitat preference (Salazar et al., 2015; Schmidt et al., 2016). In explaining these observations, we should consider cell size as a major factor that may influence relative contributions to the community RNA vs. DNA pool. While we lack culture collections for many freshwater taxa, most freshwater *Actinobacteria* are indeed very small (Hahn et al., 2003; Newton et al., 2011). Thus, *Actinobacteria* can carry a lower maximum number of ribosomes relative to the average freshwater bacterial cell. This physical trait may explain our observations, as well as previous observations of low RNA:DNA ratios for members of this phylum (Jones and Lennon, 2010; Campbell and Kirchman, 2013). Similarly, other ubiquitous and abundant freshwater taxa with low RNA:DNA ratios in our study, such as LD12 and LD28 are known to be small (Salcher et al., 2011, 2015). The low ribosomal RNA:DNA ratios for PA *Planctomycetes* seems to conflict with their relatively large cell sizes. However, this phylum is marked by extensive intracellular compartmentalization that can reduce the ribosome-containing space within the cell (Fuerst, 2005).

Can we interpret low ribosomal RNA:DNA ratios as dormancy? Based on microscopic methods, up to 80% of all aquatic bacteria have been suggested to be dormant (Cole, 1999). This is hypothesized to be a consequence of selective feeding of active cells by nanozooplankton. However, interpreting low ribosomal RNA:DNA ratios as indicative of dormancy, for example by using a threshold of “1” (Jones and Lennon, 2010), may pose some risks. First, these are ratios of relative abundance, where each value is dependent on all other populations' contributions. Second, the theoretical limit for the number of ribosomes that fit within a cell scales with cell size. Thus, we could expect small cell size to result in low RNA:DNA ratios. Despite these reservations, the relatively low growth rates for *Actinobacteria* that have been observed in *in situ* experiments support the notion that low RNA:DNA ratios correspond to low growth potential (Šimek et al., 2006). Yet, smaller cell size can confer a benefit compared to larger cells for the uptake of dissolved nutrients. Data in support of average to high contributions to substrate uptake relative to the whole community have been provided for both acI and LD12 lineages (Eckert et al., 2012; Salcher et al., 2013). Therefore, small cells may make more important contributions to nutrient cycling than we would infer based on their low ribosomal RNA:DNA ratios (Friebele et al., 1978; Azam et al., 1983). Conversely, large cells contribute a disproportionately high quantity to the RNA pool relative to the DNA pool. Does this mean they are more important to ecosystem functioning? In support of this assertion, Musat et al. (2008) observed that a bacterial population with large cell volumes that represented only 0.3% of all cells in a freshwater lake contributed >40% of all ammonium uptake and 70% of all carbon uptake in the system. Similarly, aerobic anoxygenic phototrophic (AAP) bacteria that have a 50% larger cell size than average bacteria exhibited increased levels of activity in a marine estuary (measured by leucine incorporation) (Stegman et al., 2014). Similar AAP populations contributed a disproportionately high amount of the mass flux to grazing zooplankton relative to their abundance (Garcia-Chaves et al., 2015), which again supports the greater importance of larger bacteria to ecosystem mass and energy fluxes.

Independent of our interpretation of ribosomal RNA:DNA ratios, the high similarity of RNA:DNA ratios in all spring samples is interesting. Similarity in both community composition and RNA:DNA ratios across the water column are a logical reflection of the more uniform conditions during the spring mixed period. Ribosomal RNA:DNA ratio similarity is also higher in spring between Muskegon Lake and Lake Michigan, which reflects more similar community composition at that time of the year, particularly between Muskegon Lake and nearshore Lake Michigan (Fujimoto, submitted). Similarly, RNA:DNA ratios in spring for the offshore FL samples resembles all other spring RNA:DNA ratios, despite the higher similarity of DNA and RNA community composition to lake Michigan samples taken at other times of the year (Figures 2, 5). The high similarity between RNA:DNA ratios across PA and FL fractions is the more surprising finding considering the large differences in community composition observed in this and many previous studies (e.g., Ghiglione et al., 2007; Rösel et al., 2012; Bižić-Ionescu et al., 2015; Salazar et al., 2015; Schmidt et al., 2016). While FL populations tend to outnumber PA populations (Azam et al., 1983; Ghiglione et al., 2007; Bižić-Ionescu et al., 2015), PA communities can contribute disproportionately to overall bacterial activity (Ghiglione et al., 2007; Grossart et al., 2007). As such, determining the differences in community composition and ribosomal RNA:DNA ratios of these numerically less prominent, but potentially disproportionately active populations is important.

While we cannot confidently infer bacterial contributions to ecosystem processes from ribosomal RNA:DNA ratios, our analyses identified several intriguing patterns that deserve follow-up. In particular, the seasonal changes in habitat-dependent ribosomal RNA:DNA ratios pose the question: to what extent are bacterially mediated ecosystem processes more similar across all habitats in spring? This is particularly pertinent for communities occupying the particulate and free-living fractions of freshwater lakes, as many studies in recent years have described large differences in community composition across these habitats. Future studies will need to extend our findings beyond the marker gene level, either through activity assays or by characterization of the transcriptome of the populations driving the seasonal shifts in ribosomal RNA:DNA ratios observed here.

## Author contributions

VD designed the study, analyzed data, and was the primary author of the manuscript; MF analyzed data and contributed to the writing of the paper; MB and MS provided new analytical tools, analyzed data, and contributed to the writing of the manuscript.

## Funding

VD was supported by the Community Sequencing Program (U.S. Department of Energy Joint Genome Institute, a DOE Office of Science User Facility, supported under Contract No. DE-AC02-05CH11231) and funds from the Fred A. and Barbara M. Erb Family Foundation and the University of Michigan Water Center. MS was supported by the National Science Foundation Graduate Research Fellowship (GRFP) program.

### Conflict of interest statement

The authors declare that the research was conducted in the absence of any commercial or financial relationships that could be construed as a potential conflict of interest.
